# Mutations of the Electron Transport Chain Affect Lifespan and ROS Levels in *C. elegans*

**DOI:** 10.3390/antiox14010076

**Published:** 2025-01-10

**Authors:** Fanni Ősz, Aamir Nazir, Krisztina Takács-Vellai, Zsolt Farkas

**Affiliations:** 1 Department of Biological Anthropology, Eötvös Loránd University, Pázmány P. stny. 1/C, H-1117 Budapest, Hungary; oszfanni@student.elte.hu (F.Ő.); zsolt.farkas@ttk.elte.hu (Z.F.); 2 Laboratory of Functional Genomics and Molecular Toxicology, Division of Toxicology, CSIR-Central Drug Research Institute, Lucknow 226031, India; anazir@cdri.res.in

**Keywords:** ETC, electron transport chain, SDH, succinate dehydrogenase, ROS, reactive oxygen species, *C. elegans*, cancer, lifespan, healthspan

## Abstract

Mutations in highly conserved genes encoding components of the electron transport chain (ETC) provide valuable insights into the mechanisms of oxidative stress and mitochondrial ROS (mtROS) in a wide range of diseases, including cancer, neurodegenerative disorders, and aging. This review explores the structure and function of the ETC in the context of its role in mtROS generation and regulation, emphasizing its dual roles in cellular damage and signaling. Using *Caenorhabditis elegans* as a model organism, we discuss how ETC mutations manifest as developmental abnormalities, lifespan alterations, and changes in mtROS levels. We highlight the utility of redox sensors in *C. elegans* for in vivo studies of reactive oxygen species, offering both quantitative and qualitative insights. Finally, we examine the potential of *C. elegans* as a platform for testing ETC-targeting drug candidates, including OXPHOS inhibitors, which represent promising avenues in cancer therapeutics. This review underscores the translational relevance of ETC research in *C. elegans*, bridging fundamental biology and therapeutic innovation.

## 1. Introduction

About 1 in 5000 adults suffer from primary mitochondrial diseases [1]. Most defects are due to either mutations in the nuclear genome or its mitochondrial equivalent (mtDNA), and most of these lie within the electron transport chain (ETC) [2]. Interestingly, mtDNA is prone to mutation—firstly, because it lacks wrapping histones and, secondly, because mtDNA repair is less efficient. This vulnerability is compounded by the proximity of mtDNA to the production of reactive oxygen species (ROS) from the ETC, leading to a 10- to 20-fold higher mtDNA mutation rate relative to nuclear DNA [3].

The components of the ETC are highly conserved from yeast to human [4], making the animal models of human genetic mutation tractable and attractive options to unravel the complex background of mitochondrial diseases, providing clues to possible therapeutic strategies [5,6].

Herein, we summarize knowledge about the structure and function of ETC complexes and give an overview of human diseases related to mutations of different complex subunits. Our focus is on homologous ETC mutations in the nematode *Caenorhabditis elegans* and their possible contributions to a better understanding of ETC functions. The ETC is a major source of ROS (mtROS) production in cells. By summarizing current views on redox balance and redox signaling, we demonstrate how *C. elegans* ETC mutant phenotypes are related to ROS. Our interest in this field was prompted by modelling complex II mutants by generating a clinically relevant SDHB mutant model in worms. As SDH germline mutations predispose to rare neuroendocrine tumors, we became interested in drug candidates targeting OXPHOS (thereby influencing ROS) as emerging therapeutic options in cancer treatment. Finally, we demonstrate how the nematode can be used as a test bed in evaluating effects of ETC-targeting agents.

## 2. Structure and Function of the Electron Transport Chain (ETC) Complexes and Human Diseases Related to ETC Subunit Mutations

**Figure 1:**
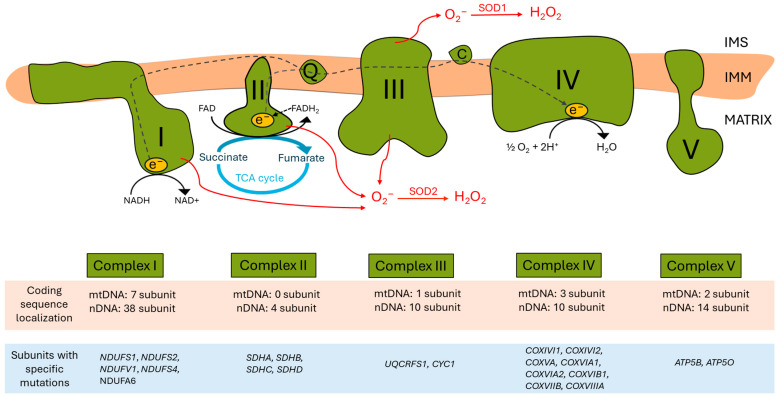
Human mitochondrial electron transport chain (ETC) complexes and the oxidative phosphorylation system. In the upper part, the four complexes of the ETC and the ATP synthase within the mitochondrial inner membrane (IMM) are schematized, alongside the electron pathway (grey dashed arrows). The intermembrane space (IMS) is at the top, and the mitochondrial matrix is at the bottom. Complexes I and II are the two main entries of reducing equivalents in the ETC. Complex I transfers electrons from NADH to CoQ (ubiquinone) in the IMM, and during the process, 4H^+^ ions are translocated into the IMS. Complex II is the SDH complex, which transfers electrons from succinate, a tricarboxylic acid (TCA) cycle intermediate, to CoQ, as well as complex I. CoQ transfers its electrons derived from complexes I and II to complex III through the phospholipid bilayer. Complex III contributes to the electron transfer from CoQ to cytochrome C (C) in the IMS; during the process, another four H^+^ ions are pumped into the IMS. Cytochrome C is a hydrophilic electron transporter that can move in the intermembrane space and transfers electrons to complex IV. Complex IV, the terminal ETC component, allows for electron transfer from cytochrome C to oxygen as a terminal electron acceptor; reduced oxygen with two H^+^ ions results in H_2_O production on the matrix side. At the same time, the complex pumps two H^+^ ions to the IMS. Complex V/ATP synthase uses the proton gradient generated across the IMM for ATP synthesis. At some points of the ETC (complexes I, II, and III), electron leakage can occur, leading to the generation of reactive oxygen species (ROS) (red arrows). In the lower part of the figure, a table summarizes the number of subunits of each complex coded either in mitochondrial DNA (mtDNA) or in nuclear DNA (nDNA) (highlighted in light brown), as well as a brief list of corresponding human subunits carrying specific mutations (highlighted in light blue).

The ETC, located in the inner mitochondrial membrane (IMM), ensures that electrons from reduced co-enzymes NADH and FADH_2_ generated during catabolic processes (also in the TCA cycle) are passed down an electrochemical gradient to elemental oxygen, the terminal electron acceptor, whilst *pari passu* drives protons outwardly into the intermembrane space. Proton re-entry across the IMM then drives ATP production [7] (Figure 1).

### 2.1. Complex I: NADH Oxidoreductase

Complex I of the electron transport chain is NADH oxidoreductase, which oxidizes NADH derived from the TCA cycle to NAD^+^ by directing the transfer of one electron pair from NADH to ubiquinone. The electron pair from NADH is first shuttled on a flavin-mononucleotide (FMN) to NADH dehydrogenase, and subsequently, inside complex I, electrons travel through Fe-S clusters to ubiquinone, which eventually becomes reduced to ubiquinol. As a consequence, four H^+^ ions are pumped into the intermembrane space.

Complex I, the largest complex of the respiratory chain, consists of 38 nuclear-encoded subunits and 7 mitochondrially encoded partners [8,9]. Complex I is L-shaped, with a hydrophilic peripheral arm protruding into the matrix, while the hydrophobic partner is embedded in the IMM. The peripheral arm contains the redox centers, the NADH-oxidizing N module and the Q module, which reduce ubiquinone. The proton-translocating machinery (P module) is localized in the membrane arm [10,11]. Fourteen conserved core subunits of complex I are necessary and sufficient for the catalysis of energy transduction: half of the core subunits belong to the peripheral arm, and the other half belong to the membrane arm. Thirty-one additional (supernumerary) subunits are distributed around the core; these subunits are functionally less well characterized but likely contribute towards the control of the assembly and the stability of the complex [12].

Most patients with primary mitochondrial diseases have complex I mutations [13]. Complex I deficiencies represent one-third of all early-onset mitochondrial disorders, with mutations in both nuclear and mitochondrial genes generating a wide range of clinical outcomes. The clinical phenotypes are classified into five groups as follows: Leigh syndrome, progressive leukoencephalopathy, neonatal cardiomyopathy, severe infantile lactic acidosis, and a group of unspecified encephalomyopathies [14].

Nuclear-encoded *NDUFS1*, *NDUFS2*, *NDUFV1*, and *NDUFS4* genes are mutational hot spots for isolated complex I deficiency [8]. For example, numerous mutations (over 40) have been identified in N-module subunit NDUFV1, resulting in various phenotypes, such as hypotonia, lethargy, myopathy, or fatigue [15]. Substitutions of E104A, M292T, R118Q, M443K, E148K, and F84L in the NDUFS2 subunit in the Q module lead to Leigh syndrome [16,17], while R138Q, R333Q, and M292T mutations of NDUFS2 result in a Leigh-like syndrome [17]. Patients with R228Q, P229Q, and S413P mutations in NDUFS2 suffer from cardiomyopathy and encephalomyopathy [18]. N24A and R30A substitutions in supernumerary subunit NDUFB4 disrupt supercomplex assembly [19]. A recent report described neurological symptoms and/or elevated lactate levels in the cases of four unrelated children with various substitutions and/or deletions in the NDUFA6 gene, either in a homozygous or heterozygous state. Symptoms arose after 3 months and 2 years of age in the cases of two patients. The third patient died at the age of 13 weeks. NDUFA6 is a supernumerary subunit of the Q module, and investigations performed on fibroblasts of the above subjects showed complex I assembly defects [20].

Besides the structural subunits of electron transport, complex I assembly requires additional factors. The mutation of these factors can lead to structural defects of complex I. Patients carrying different mutations (deletions or substitutions of Val546Leu and Ala170Val) in complex I assembly factor ACAD9 suffer from an isolated oxidative phosphorylation complex I deficiency, affecting primarily muscles, the liver, nervous system, and heart [21,22,23,24].

### 2.2. Complex II: Succinate Dehydrogenase (SDH)

Complex II transfers electrons from FADH_2_ through Fe-S clusters to ubiquinone. The hydrophobic benzoquinone structure of ubiquinone/ubiquinol enables the transfer of electrons derived from complexes I and complex II to complex III through the phospholipid bilayer.

Mitochondrial complex II is also called the succinate dehydrogenase complex (SDH or succinate–ubiquinone oxidoreductase). A unique property of the SDH complex/complex II compared to other ETC components is that paralogous genes encoding the different subunits are located exclusively in the nuclear genome. SDH is the only complex of the respiratory chain that does not pump protons across the mitochondrial inner membrane during its active catalytic cycle. This complex plays a dual role in generating energy: in the TCA cycle, SDH oxidizes succinate to double-bonded fumarate; then, using electrons released by the succinate–fumarate conversion, it catalyzes the reduction of ubiquinone to ubiquinol in the mitochondrial respiratory chain.

The tetrameric SDH enzyme complex consists of four functionally distinct subunits, SDHA, SDHB, SDHC, and SDHD. All the four subunits are encoded by the nuclear genome. The 3D structure of the well-known porcine heart SDH shows that the enzyme complex has a hydrophilic head protruding into the mitochondrial matrix and a hydrophobic tail located within the mitochondrial inner membrane (IMM) [25,26].

Hydrophilic SDHA and SDHB subunits, together, form the catalytic site of the enzyme. SDHA is a flavoprotein containing the binding site for succinate and a covalently bound FAD prosthetic group. SDHB is a subunit displaying three strongly conserved iron–sulfur clusters ([2Fe-2S], [4Fe-4S], and [3Fe-4S]), which undergo oxidation-=–reduction processes as the electrons pass. Hydrophobic SDHC and SDHD subunits anchor the enzyme complex to the IMM and contain cytochrome-B [25,27].

The SDH enzymatic reaction starts with the binding of succinate to its binding site, subsequently leading to a conformational change in the SDHA subunit and oxidation of succinate with FAD, which, in turn, becomes reduced to FADH_2_. Next, electrons from FADH_2_ pass through the one electron-carrier Fe/S centers and, finally, convert/reduce ubiquinone to ubiquinol in two successive steps.

Mammalian SDH complexes display two ubiquinone binding sites: the proximal site (QP) is located closer to the matrix, at the interface of SDHB, SDHC, and SDHD subunits, and shows higher affinity for ubiquinone [25]. The other, distal ubiquinone binding site (QD) with lower affinity for ubiquinone is more distant from the matrix [27]. The importance of the QD site and the conserved haem moiety remains to be determined in eukaryotes [27].

The assembly of the SDH complex has recently been understood and requires the coordinated action of four assembly factors, which are necessary for the maturation of the soluble SDHA and SDHB subunits and subsequent assembly of the entire complex [28].

Germline mutations in the four SDH subunits of complex II predispose to various tumors: paragangliomas, pheochromocytomas, and gastrointestinal stromal tumors (GIST) [29]. Pheochromocytomas (PHEOs) and paragangliomas (PGLs) are rare neuroendocrine tumors arising from the adrenal medulla or from sympathetic and parasympathetic ganglia of the peripheral nerve system, respectively [30]. SDH-derived GISTs make up approximately 10% of these tumors [31], which are mainly formed in the stomach, and patients are usually younger than 30 years old [32]. SDH-derived paragangliomas can also present in dyads with GISTs (Carney-Stratakis syndrome) or in triads with GISTs and pulmonary adenoma (Carney triad). SDH-mutated PGLs/PHEOs have also been reported with pituitary adenomas. SDH-deficiency also occurs in 0.05–0.2% of renal cell carcinomas [33].

Many substitutions have been described in the SDHB subunit that are associated with GIST, renal cell carcinoma, multiple hamartomas, acute T-cell leukemia, PHEOs, and PGLs (reviewed in [34,35]). Some of them display regional localization, like the R46Q Maori mutation [36,37,38]. Germline mutations in the *SDHC* gene can also lead to PGL [39]. Some SDHA mutations, such as L511P, G233V, Arg31*, Arg512*, S445L, and UTRdel, cause PHEO and PGL [40], while other SDHA germline mutations have been reported in SDH-deficient GISTs [41].

**CoQ** (**coenzyme Q**/ubiquinone/ubiquinol) has a benzoquinone head group and a long isoprenoid side chain and resides in biological membranes [42]. In the ETC, CoQ acts as a transmitter of electrons from complexes I and II to complex III. CoQ7 mutations (for example, R54Q) result in a decrease in coenzyme Q10 production [43] and manifest in different clinical phenotypes. 1Met? mutation (c.3G > T (p.1Met?) mutation changes the canonical ATG start codon into an ATT sequence, blocking the initiation of translation, which leads to distal hereditary motor neuropathy [44], and Ala205HisfsTer48 and Met135Val cause cardiomyopathy and gastrointestinal obstruction [45], while Pro108Thr results in hereditary spastic paraplegia [46]. The increasing number of case reports describing different CoQ mutations [47,48] might provide clues to possible treatments.

### 2.3. Complex III: Cytochrome C Reductase

Complex III or cytochrome C reductase constitutes the central component of the ETC. The structure forms a symmetric dimer [49]; each of the monomers is composed of 11 different subunits [50]. Of the three proton-pumping complexes, complex III, has the fewest subunits: one encoded by mtDNA and 10 encoded on nDNA [50]. Complex III contains a series of catalytic subunits: cytochrome b, containing two CoQ binding sites and two heme *b* groups; UQCRFS1, the Rieske Fe–S protein; and CYC1, containing heme *c* as the prosthetic group. All of these contribute to electron transfer from ubiquinol (CoQ) through cytochrome C to complex IV whilst also contributing to the generation of the proton gradient across the mitochondrial membrane. An interesting step of complex III assembly is the post-translational cleavage of an N-terminal fragment of the UQCRFS1 protein known as ‘UQCRFS1N’. Cleavage is undertaken by the matrix processing peptidase activity of UQCRC1 and UQCRC2 [51]. UQCRFS1N is retained and bound in the interface between the UQCRC1 and UQCRC2 subunits [52].

UQCRFS1 mutants show a reduction in mitochondrial complex III activity. Homozygous Val72_Thr81del10, in combination with Val14Asp/p.Arg204*, leads to cardiomyopathy and alopecia totalis [53,54]. Trp96Cys and Leu215Phe mutations in human CYC1 manifest in insulin-responsive hyperglycemia [55].

**CyCS** (**cytochrome C**) shuttles electrons between complexes III and IV. Both Lys101del mutation in the α-helix of the C-terminal domain of CYCS and His27Tyr substitution result in autosomal dominant non-syndromic thrombocytopenia [56,57]. G41S, Y48H, and Tyr98His substitutions cause thrombocytopenia and affect cellular bioenergetics and apoptosis [58,59,60].

### 2.4. Complex IV: Cytochrome C Oxidase

Complex IV of the mitochondrial respiratory chain (also called cytochrome C oxidase; COX) (Figure 1) is composed of 13 subunits that can be divided into two groups: subunits I–III are the catalytic subunits of COX and are encoded in the mitochondrial genome, while subunits IV, VA, VB, VIA, VIB, VIC, VIIA, VIIB, and VIII are the so-called supernumerary subunits that are encoded in the nuclear genome [61].

Subunit II transfers electrons from cytochrome C to subunit I via its CuA site, composed of two copper atoms. The electrons are then transferred to the heme a group of subunit I and subsequently to the oxygen-binding site of subunit I, composed of heme a3, CuB, and a tyrosyl group. Four electrons are transferred simultaneously to the electron acceptor O_2_, which prevents the formation of ROS. Subunit III is responsible for the stabilization of the catalytic center and for proton pumping [62]. Less is known about the role of the supernumerary subunits, and it has been proposed that they have structural roles.

COX activity can be regulated through subunit IV. Depending on the ATP/ADP ratio of the mitochondrial matrix, subunit IV binds either ADP or ATP at its matrix domain. The conversion of bound ADP to ATP leads to the feedback inhibition of COX [63]. Other regulatory factors of COX include metabolite and protein binding, expression of supernumerary subunit isoforms, phosphorylation, and the formation of supercomplexes [62]. Mutations in complex IV structural subunits, assembly factors, or cofactor synthesis can lead to a variety of diseases, such as Leigh syndrome, Fanconi anemia, Charcot–Marie–Tooth syndrome, and neurological disorders (for a more comprehensive list, please see [29]). The K101N mutation in COXIVI1 leads to poor weight gain, short stature, and an increase in chromosomal breaks, resembling Fanconi anemia [64]. The P152T mutation of COXIVI1 causes clinical features resembling Leigh syndrome [65]. The E138K mutation in COXIVI2 causes calvarial hyperostosis, dyserythropoietic anemia, and exocrine pancreatic insufficiency [66]. The R107C mutation in COXVA presents with lactic acidemia, failure to thrive, and pulmonary arterial hypertension [67]. A 5 bp long deletional mutation of COXVIA1 causes the muscle wasting/neurological disease known as Charcot-Marie-Tooth disease [68], while the S39R substitution in COXVIA2 leads to muscle weakness [69]. The R19H mutation in COXVIB1 causes mitochondrial encephalomyopathy [70], while the R20C mutation in COXVIB1 not only causes encephalomyopathy but also cardiomyopathy and hydrocephalus [71]. Different COXVIIB mutations have been identified to cause microphthalmia with linear skin lesions [72]. Loss of COXVIIIA leads to Leigh-like syndrome and epilepsy [73].

### 2.5. Complex V: ATP Synthase

Complex V, also known as ATP synthase, plays a pivotal role in energy production within the cell via synthesis of ATP using the proton gradient across the inner mitochondrial membrane (IMM). This enzyme complex consists of multiple protein subunits divided into two main regions: the hydrophobic F_0_ and the hydrophilic F_1_ regions [74]. The F_0_ region, embedded within the IMM, includes a proton channel, which is crucial for the translocation of H^+^ ions. These ions are known to be essential for the mechanical rotation necessary for ATP synthesis. Key components of the F_0_ region include subunits a, b, and c, which form the proton channel [75]. The F_1_ portion is exposed to the mitochondrial matrix and houses the enzyme’s catalytic activity. It consists of five subunits (α3, β3, γ, δ, and ε) arranged in a complex structure. The gamma subunit acts as a central shaft surrounded by alternating alpha and beta subunits, which, together, form a hexameric ring. As protons move through the F_0_ region and enter the F_1_ region, they induce rotational motion in the gamma subunit, which, in turn, drives the cyclic interactions of ADP and inorganic phosphate (Pi) with the beta subunits, catalyzing ATP synthesis [76]. Under certain conditions, when mitochondrial electron transport is interrupted (after inhibition of the electron transport chain or exposure to uncouplers), ATP synthase can run in reverse by hydrolyzing ATP and transferring protons from the matrix to the intermembrane space, thereby building the necessary Δp (proton motive force) and maintaining membrane potential. This process consumes ATP, and there exists a defense mechanism against ATP depletion mediated by the mitochondrial ATPase. This mechanism requires endogenous inhibitor protein ATPIF1, which binds to the ATPase when the matrix pH falls (reviewed in [77,78]). Importantly, a polyphenolic compound, (+)-epicatechin (EPI), appears to selectively inhibit the ATPase activity of complex V without affecting ATP synthase activity [79].

*ATP5B* and *ATP5O* are significant genes associated with human ATP synthase. *ATP5B* encodes a subunit of the F_1_ region, specifically one of the beta subunits crucial for enzymatic activity. Mutations in *ATP5B* have been linked to various mitochondrial disorders, affecting ATP synthesis efficiency and influencing cellular energy metabolism [80,81]. *ATP5O*, encoding the O subunit (also known as OSCP: oligomycin sensitivity conferral protein) of ATP synthase, is part of the structural stator linking the F_0_ and F_1_ regions. It is crucial for stabilizing the interface between these enzyme sections. In humans, mutations in ATP5O have not been reported commonly but are thought to impact the assembly and stability of the ATP synthase complex, leading to diseases such as those of neuromuscular and cardiac systems.

All mutations in different ETC complex subunit genes discussed above are listed in Table 1.

## 3. Redox Signaling: Past and Present Research

The term reactive oxygen species (ROS) includes non-radical H_2_O_2_ (hydrogen peroxide) and its radical derivatives (i.e., containing an unpaired valence electron such as •HO_2_ (hydroperoxyl radical), also known as the hydrogen superoxide, **^.^**HO•: hydroxyl radical, OH^−^: hydroxyl anion, ^1^O_2_•: singlet oxygen, and O_2_**^−^**: superoxide) [82]. Based on their reactivity, ROS can be grouped into two categories: (1) low-reactivity ROS and (2) high-reactivity ROS. Superoxide and hydrogen peroxide, the most abundant and best studied ROS in vivo [83] are low-reactivity ROS. Free-radical superoxide and the non-radical hydrogen peroxide are produced when oxygen is reduced with one or two electrons, respectively. Low-reactivity ROS have a limited capacity to damage macromolecules [84], as specific enzymatic systems of antioxidants neutralize them in the cell. Superoxide is converted to hydrogen peroxide by superoxide dismutases [84]; hydrogen peroxide is detoxified by other types of enzymes, such as catalases, glutathione peroxidases, and peroxiredoxins. In contrast, high-reactivity ROS (for example, the hydroxyl radical) are harmful, as they directly attack macromolecules; in addition, cells lack specific antioxidant systems to eliminate them.

Mitochondrial ROS (mtROS), including superoxide, hydrogen peroxide, and the hydroxyl radical derived from complex I (CI), complex II (CII), and complex III (CIII) of the ETC [85,86], are the most abundant source of reactive oxygen species in the cell, but ROS are also produced in peroxisomes during fatty acid β-oxidation and in the endoplasmic reticulum (ER) during disulfide bond formation of protein folding [87]. The ER, mitochondria, and peroxisomes are often called ‘the redox triangle’; we note that they are in direct contact with each other through membrane contact sites and generate and transport oxidants into the cytoplasm. In addition, essentially all cellular organelles communicate via redox signaling [88].

Past research identified mtROS as toxic agents causing oxidative damage to biological molecules, thereby contributing to age-related diseases. In the last two decades, we have learned that mtROS produced in specific places at specific times and intensities act as signaling molecules and determine the downstream effects of mitochondrial redox signaling, which is essential to maintain cellular homeostasis [89].

Technologies to measure different ROS have undergone significant progress in the last decade [90]. While early measurements of mtROS were performed on isolated mitochondria in vitro, recently, fluorescent probes (either small dyes or genetically encoded fluorescent reporters) have been developed to allow for in vivo measurements [91]. *C. elegans* also contributes to measurement of some types of reactive oxygen species in vivo. Using H_2_O_2_ redox sensor HyPer under the control of a ubiquitous promoter, it is possible to determine the tissue/cell type and the developmental stage where hydrogen peroxide is produced [92]. Grx1-roGFP1 and Grx1-roGFP2 probes detect the GSSG/2GSH ratio, which monitors the glutathione redox potential in vivo [92]. These redox sensors directly or indirectly provide quantitative and qualitative (temporal and spatial) information about the production of given reactive oxygen species in *C. elegans*, adding to the tractability of this organism.

### 3.1. Past Research Focused on Investigation of Defense Against Oxidative Stress Mechanisms That Eliminate Excess ROS

As elevated levels of ROS have been implicated in the pathogenesis of cancer, neurodegenerative disorders, and cardiovascular diseases (age-related disorders) [93], investigations in the past focused on harmful effects of ROS, i.e., the oxidative stress caused by increased ROS levels. ROS and other free radicals were thought to be byproducts of cellular metabolism [94]. Due to their capacity to cause oxidative damage, mtROS were considered main drivers of aging [83]. The goal has been to seek out signaling pathways that drive homeostasis, protect cells against oxidative damage, and eliminate ROS. Research has identified three key players as ROS-regulated transcription factors: NRF2 (nuclear factor erythroid 2-related), NF-κB (nuclear factor kappa-light-chain enhancer of activated B cells), and p53. Classically, NRF2 is responsible for cell homeostasis by eliminating DNA-damaging agents, including ROS. NRF2 binds to antioxidant response elements (AREs) of its target genes, such as HMOX1 (heme oxygenase), GCL (glutamate-cysteine ligase), NQO1 (NAD(P)H quinone oxidoreductase 1), and GSTs (glutathione S transferases). Keap1 (Kelch ECH-associating protein 1) is the repressor of NRF2 that promotes its degradation [95]; however, elevated ROS lead to the oxidation of Keap1, which prevents the degradation of NRF2, thereby resulting in NRF2 activation [96]. The regulation of p53 by ROS differentially depends on ROS concentrations: low ROS prompt the upregulation of p53-activated antioxidant genes, while in the case of a higher ROS level, p53 activates pro-oxidant genes, inducing cell death [97]. Such data point to a new approach involving terms such as eustress and hormesis, as discussed below.

*C. elegans* also possesses an evolutionarily conserved protective system to deal with harmful increases in ROS [98]. Phase II detoxification enzymes are present in the nematode [98].

In the worm intestine, which is often the first tissue facing pathogens and xenobiotics, ROS-induced activation of detoxifying genes is mediated by NRF homolog SKN-1, which acts as a transcription factor through ARE binding [99]. Indeed, ROS-protecting enzymes in the gut may be sufficient for *C. elegans* stress resistance [100,101]. Upon oxidative stress, SKN-1 is activated via a conserved p38 MAP kinase cascade, which leads to PMK- 1/MAPK-mediated phosphorylation and nuclear localization of SKN-1, as well as subsequent induction of stress-responsive genes [102]. This implies a regulatory interface between ROS and protein phosphorylation.

Other transcriptional regulators are also involved in the activation of ROS-protective gene expression in worms. Among them, the FOXO homolog DAF-16 transcription factor plays an important role. Although DAF-16 has different target genes from SKIN-1, they share similar regulatory mechanisms; DAF-16 is also activated through the stress-activated p38 MAPK pathway [103]. Under normal, stress-free growth conditions, both DAF-16 and SKN-1 are phosphorylated on specific residues by kinases of the insulin/insulin-like signaling pathway, resulting in DAF-16 and SKN-1 retention in the cytoplasm, inhibiting their translocation to the nucleus [104,105]. Whether ROS levels interfere with this phosphorylation remains to be determined.

Importantly, the activity of master transcription factors DAF-16 and SKN-1 is essential in a wide range of stress responses and is required for longevity [98,106,107].

### 3.2. The Current View: Redox Flux—Old Lamps for New Flammation via Eustress to Hormesis

Although excess ROS cause oxidative damage to biomolecules, it has become clear that a lack of appropriate oxidant levels also impairs signaling events and damages cellular processes. Recent evidence suggests that ROS, if expressed with the correct concentration and sub-cellular localization, can be beneficial and act as signaling molecules, regulating different physiological processes (reviewed in [108]).

Cells maintain a delicate balance between the level of oxidants and regulatory antioxidants. If the redox balance is shifted towards oxidants, oxidative distress occurs that favors antioxidants, potentially leading to reductive distress. Thus, an emerging idea is that the levels oxidants and antioxidants are balanced by redox regulation (reviewed in [109]). Redox regulation involves redox sensing, redox signaling, and subsequent signal translation to cellular stress responses. Stress responses cause changes in the activity of redox-regulated proteins and influence gene expression by modifying the epigenetic landscape. For example, SIRT1/sirtuin is under redox regulation, and its oxidation results in inactivation of its deacetylase function [110]. Oxidation reactions are counterbalanced by reduction reactions using NADH and NADPH. Briefly, NADH/NADPH is linked to sulfur metabolism through thioredoxin and glutathione (GSH) systems, which are necessary to maintain the redox state of proteins and are the primary targets of redox modifications in cells. Redox protein modifications occur on sulfur- and selenium-containing constituents of proteins (e.g., cysteine, methionine, selenocysteine, and selenomethionine residues). *C. elegans* studies (below) demonstrate how redox modification on given cysteine residues of specific proteins can lead to different biological responses.

For example, ROS can directly sulfenylate a cysteine residue within the ER stress sensor IRE-1 kinase, which results in activation of the p38 MAPK/SKN-1 axis, thereby increasing stress resistance and lifespan in *C. elegans* [111]. A recent study showed that mitochondrial superoxide enhances an RAS-dependent ROS signaling pathway called RDRS that controls the expression of a large set of genes regulating a variety of cellular functions with enhanced RDRS signaling in response to mtROS that results in lifespan extension [112]. Superoxide is converted by superoxide dismutase SOD-1 into cytoplasmic hydrogen peroxide, yet oxidizes a cysteine residue (C118) of LET-60/RAS. Hydrogen peroxide generated by redox pathways in different cellular compartments serves as a second messenger and has a major role in maintaining redox homeostasis [88,109].

In summary, the maintenance of redox homeostasis requires constant monitoring and reprogramming of redox fluctuations. The dynamic turnover of oxidative metabolism is a continuous challenge, resulting in a low level of oxidative stress (eustress) operating within a physiological range. Low levels of the oxidant species lead to cell adaptation to stress that protects against higher levels of oxidative stress; this is the principle of hormesis [113,114].

### 3.3. Complexities of ROS Signaling in Normal Health Conditions and Disease

In this chapter, we highlight some examples of how ROS signaling contributes to normal cellular functions and how cellular ROS levels can be targeted for therapeutic use.

ROS are accumulated during reverse electron transport (RET) in a site-specific way in respiratory complex I. A significant amount of ROS generated by this process (also called RET-ROS) is necessary for several cellular functions, for example, macrophage activation in response to bacterial infection.

Targeting ROS levels is also a developing approach to treat diseases. For example, increasing ROS in cancer cells to toxic levels in order to kill them by apoptosis is the principle of pro-oxidant therapies, as we discuss below.

#### 3.3.1. RET-ROS Signaling in Health and Disease

The process of reverse electron transport (RET) was first described in 1961 [115], when the addition of succinate into isolated mitochondria resulted in the redirection of electrons from ubiquinol to complex I and the reduction of NAD+ to NADH by the NADH dehydrogenase module of complex I [116]. RET is an energetically unfavorable reaction that only occurs when there is a high ubiquinol/ubiquinone ratio and high proton motive force (Δp) in the mitochondria. During RET, NAD+ is reduced at the expense of ATP, and protons are pumped from the matrix to the intermembrane space. RET is also a major source of ROS in cells [116]. ROS generated during RET (RET-ROS) has been shown to initiate an anti-inflammatory response of macrophages after bacterial infection. ROS are required to produce anti-inflammatory cytokines in macrophages. Note that in order to boost ROS levels, macrophages shift their metabolism from OXPHOS to glycolysis to generate ATP and increase succinate oxidation by complex II/SDH with an elevated mitochondrial membrane potential, which, in turn, leads to RET and RET-ROS production [117]. Interestingly, RET-ROS signaling was blocked by ectopic expression of an alternative oxidase (AOX) derived from *Ciona intestinalis* in mice. AOX prevents the over-reduction of CoQ [118] and inhibits the inflammatory phenotype of mouse macrophages [117]. Another beneficial role of RET-ROS signaling was reported in the adaptation of the carotid body to hypoxia [119]. In contrast, deregulated RET-ROS has been identified as a main factor causing stroke, and decreases in the NAD^+^/NADH ratio induced by RET have been linked to many age-related disorders, such as neurodegeneration and cancer (reviewed in [120]).

#### 3.3.2. Targeting ROS in Cancer

Targeting ROS for therapeutic benefit involves either reducing excessive ROS to alleviate oxidative damage or increasing ROS to cytotoxic levels to kill cancer cells. Antioxidants such as N-acetylcysteine (NAC), vitamin C, and vitamin E are commonly investigated for their potential to scavenge ROS and protect against oxidative stress in various diseases. However, the clinical efficacy of antioxidants in cancer therapy has been mixed, with some studies suggesting that antioxidants might protect cancer cells from ROS-induced cytotoxicity [121].

On the other hand, pro-oxidant therapies aim to exploit the elevated basal ROS levels in cancer cells. Agents such as arsenic trioxide, menadione, and elesclomol selectively induce lethal levels of ROS in cancer cells. This therapeutic strategy is based on the concept that cancer cells, already under oxidative stress, are closer to the threshold of ROS-induced apoptosis, making them more vulnerable to further ROS insults [122].

## 4. Effects Exerted by Mutations in ETC Genes on Development and Lifespan of Nematodes

As humanity gained a high level of medical and technical knowledge, our research turned towards enhancing not only the quantity but also the quality of our extended life. This is a challenging task, as in most cases, advancing age (longer lifespan) is accompanied by reduced well-being (as known as healthspan) [123]. While lifespan is the total number of years of an individual’s life, healthspan is that period which is free from disabilities and/or chronic diseases. These two dimensions are closely bonded and cannot be discussed without the inspection of the phenomenon called aging, which is affected by various factors, like genetic, cellular, and environmental features. But one of the key drivers of aging is the decay of mitochondrial function, causing energetic decline at the cellular level [123]. Mitochondrial dysfunction has a crucial impact on lifespan, as well as on healthspan. Therefore, investigating the consequences of mitochondrial dysfunction in a variety of complex model organisms is vital in developing new strategies to improve quality of life in our aging society [123].

With its many advantages—e.g., short life cycle and 65–70% genome sequence homology to humans—*Caenorhabditis elegans* is a popular model in many aspects of developmental biology, especially in the field of aging [124]. This simple model organism is also capable of easily determining the difference between lifespan and healthspan, as standardized methods have been developed recently, like the measurement of movement, pharyngeal pumping, brood size, etc. [124,125,126]. Many characteristics of *C. elegans* and human mtDNA are similar, such as their structure and size (13.7 vs. 16.6 kb), maternal inheritance, polyploidy, and heteroplasmy [127,128]. Furthermore, most biochemical properties of worm mitochondria are similar to those of mammals, such as oxygen consumption profile; supercomplex formation; and, most importantly, TCA cycle and ETC activities [129,130]. Amongst 91 human genes encoding ETC components, 72 have orthologs in *C. elegans* [131]. The conservation rate of these subunits is significant; for instance, sequence similarity at the amino acid level between *C. elegans* and human homologous complex I subunits varies between 25% and 99.2%. These data, together, support an extensive evolutionary conservation between *C. elegans* and humans in terms of ETC structure and function [132].

### 4.1. Mutations of Complex I Subunits

Thirty-one nuclear-encoded mammalian complex I subunit genes have orthologs in *C. elegans* [132]. Homologs of *NDUFV1*, *NDUFS2*, *NDUFB4*, and *NDUFA6* have been extensively characterized in the nematode. Grad and Lemire generated transgenic worms carrying clinically relevant point mutations in the highly conserved *nuo-1* gene in *C. elegans*. A352V, T434M, and the A443F mutant versions of NUO-1 correspond to the disease-causing substitutions in the NDUFV1 protein, A341V, T423M, and A432F, respectively. These conserved residues are important for protein folding, and the mutant NUO-1 proteins seem more susceptible to degradation. All the mutants displayed decreased lifespan, low brood size, and hypersensitivity to hyperoxia and (pro-oxidant) paraquat [133]. Importantly, transgenic worms showed hallmarks of complex I dysfunction such as an increase in lactate and the lactate:pyruvate ratio (lactic acidosis) and decreased NADH-dependent mitochondrial respiration. *nuo-1*(RNAi) phenocopied lactic acidosis, reminiscent of that which is observed in some patients with mitochondrial disorders [134].

A deletional allele (*ua1*) of *NDUFV1* homolog *nuo-1* (*NADH ubiquinone oxidoreductase 1*) results in L3 larval arrest and increased lifespan [135]. L3-to-L4 transition has a high energy demand underpinned by increases in oxygen consumption [135] and mtDNA copy number. A lack of ETC subunits in *nuo-1* mutants results in severely decreased energy production that does not allow for the transition to the L4 stage.

In contrast, the *fc21* deletional allele of *NDUSFS2* homolog *gas-1* (general anesthetic sensitivity abnormal 1) shows slowed development and reduced lifespan [136]. Furthermore *gas-1* loss-of-function mutant animals are hypersensitive to volatile anesthetics [137]. This ‘worm to cot side’ discovery originated in *C. elegans* to guide clinical recommendations in mitochondrial disorder patients for proper dosing of volatile anesthetics [138]. Similarly, experiments conducted on the nematode showed that riboflavin rescued complex I stability and partially rescued complex I and IV activities in *nuo-1* point mutants [139]. Based on these data, riboflavin supplementation has been proposed as a possible therapeutic intervention to treat NDUFV1 and NDUFV2 defects, in addition to also possibly treating defects in the other flavoproteins, like NDUFV3 [140].

Another complex I mutant, *NDUFB4* homolog *nuo-6*(*qm200*), showed decreased respiration and electron transport rates, despite increases in ATP concentrations. *Nuo-6*(*qm200*)-like *gas-1*(*fc21*) and *nuo-1*(*ua1*) display slower embryonic and postembryonic development. *Nuo- 6*(*qm200*) animals have an elevated lifespan [141].

Interestingly, ETC complex I dysfunction was rescued by hypoxia in a mouse neurological disease model [142]. Recently, another group used the nematode as a model to reveal the unknown mechanism behind this finding [143]. *NDUFA6*/*nuo-3*(*G60D*), which phenocopies hypoxia rescue or hypoxia, directly restored complex I activity and rescued ETC flux and, in some cases, complex I levels (*nduf-7*(*et19*) *and gas-1*(*fc21*)). In a screen, NDUFS2/GAS-1(V161) and NDUFS7/NDUF-7(M80)—critical residues in the ubiquinone binding pocket—were identified as suppressors of *nuo-3*(*G60D*) [143]. Meisel et al. showed that these mutants also blocked the rescue of *nduf-7*(*et19*) and *gas-1*(*fc21*) by hypoxia, pointing to a shared rescue mechanism between *nuo-3*(*G60D*) and hypoxia. Results suggest that the activity of complex I mutants is rescued either by altering the conformation of the ubiquinone binding pocket, or by influencing the local chemical environment. In summary, Meisel et al. showed that hypoxia and *nuo-3*(*G60D*) rescue complex I mutants through a shared mechanism via key residues in the ubiquinone binding site [143].

Deficiency of ACDH-12 (Acyl CoA dehydrogenase), the homolog of complex I assembly factor ACAD9, affects the function and formation of complex I. RNAi knockdown of *acdh-12* shortens lifespan [144].

Knocking down any of the complex I subunits, for example, *gas-1*(*fc21*) [145], leads to increased **complex II**-dependent respiration [132].

### 4.2. Mutations of Complex II Subunits

Interestingly, SDHA and SDHB (but not C and D) subunits are evolutionarily highly conserved at the amino acid level [146]. As the SDH complex has been the focus of our work recently, we performed pairwise alignments of the relevant worm and human SDH subunit homologs, and the results of sequence alignments underpin this statement. Indeed, in *C. elegans* SDHA and SDHB subunits show higher similarity and identity to their human counterparts at the amino acid level (SDHA: 83% and 73%; SDHB: 84% and 60%, respectively) compared to SDHC and SDHD subunits, where these similarity and identity values are lower (SDHC: 54% and 30%; SDHD: 59% and 43%, respectively). Interestingly many agricultural pest control agents target the SDHx complex (complex II) [147], and some of them are being considered for use in human anti-fungal treatments [148]. However, it is important to note that many of these substances are nematicides as well; for example, fluopyram is described as a selective nematicide [149]. The SDHx complex is well characterized in the nematode; *mev-1* encodes CYT-1, cytochrome b560, which is the large subunit of succinate–ubiquinone oxidoreductase in SDH (the SDHC subunit). It was the first discovered oxygen sensitivity mutant [150]: Ishii et al. originally screened for mutants sensitive to methyl viologen (paraquat), which is a redox-active herbicide, and identified *mev-1*(*kn1*)*-* (G71E) as a paraquat-sensitive mutant. This homozygous point mutation not only disturbs the biochemical process of succinate/fumarate conversion but also shows an effect on the respiratory electron transport chain by electron leakage. *mev-1*(*kn1*) animals display a short lifespan, reduced brood-size phenotype, and oxygen hypersensitivity, while the deletional allele of *mev-1*(tm1081) is lethal [151,152]. These observations have subsequently been recapitulated in a mouse model, showing that these SDHC-related processes are evolutionarily conserved [153]. Interestingly, Pujol et al. described an elevated *gas-1*(fc21) lifespan upon *sdhc-1* and *sdhd-1* knockdown [154]. Since then, based on these traits (accelerated aging and susceptibility to oxidative stress) the *mev-1* mutants have been used frequently as a model of mitochondrial malfunction [155] and as test beds in the search for new anti-aging antioxidants [156].

The other well characterized SDH complex member in worms is B-subunit ortholog *sdhb-1*. Huang and Lemire discovered that different mutations in the *sdhb-1* gene resulted in superoxide generation and premature aging [157]. They observed impaired SDH function and assembly, increased superoxide anion production, and perturbed mitochondrial respiration due to a mutation in Pro211, a conserved proline residue—which corresponds to Pro197 in the human counterpart—near the proximal quinone binding site (Qp) in SDHB-1. Furthermore, they showed that different missense Pro211 mutations (Pro211His, Pro211Leu, Pro211Phe, and Pro211Arg) cause embryonic lethality with incomplete penetrance and shortened lifespan in the surviving animals. Huang and Lemire also showed that *sdhb-1* null mutants carrying the *gk165* deletional allele arrest development in the L2/L3 larval stage [157]. Another group studied the effect of SDH deficiency on the hypoxia system in worms [158]. One consequence of HIF (hypoxia-inducible factor) activation in worms is a defect in egg laying. To study HIF activation, Braun et al. knocked down SDHB in a subset of neurons thought to be responsible for egg laying. They found that *sdhb-1* depletion resulted in the expected excess succinate level, which inhibited prolyl-hydroxylase EGL-9. EGL-9 inhibition prevented HIF-1 hydroxylation and degradation, thereby activating HIF-1 signaling, which led, inter alia, to the retention of eggs [158].

Lately, our lab has built on these data to make a new disease model: the SDHB Arg244His mutation was introduced into the worm, which is a clinically relevant germline mutation, corresponding to Arg230His causing human paraganglioma [159]. We showed that this point mutation makes the SDH enzyme defective, with abnormal succinate accumulation and reduced oxygen consumption. It has been proposed that excess succinate levels are also characteristic of PHEO/PGL tumors, where SDH mutations cause mitochondrial succinate accumulation in the cytoplasm and HIF-1α (hypoxia-inducible factor alpha) activation, which favors pseudohypoxia-driven tumorigenesis [160] and can lead to inflammation [161]. Arg244His worms also develop abnormally, but unlike *sdhb-1* null mutants—which arrest in the L2/L3 larval stage—these point mutants reach adulthood but remain sterile and show slowed development. Our data contrast with the larval arrest of the null mutants that occurs at the L2/L3 stage, likely because loss of SDH activity is compensated for by the glyoxylate cycle, which is most active during embryonic development and peaks at the L1 larval stage, declining thereafter by the end of the L2 stage [130]. However, both *sdhb-1* deletion and Arg244His missense mutation shorten lifespan. In line with these data, silencing of *sdhb-1* by RNAi also resulted in a shortened lifespan [154]. This shortened lifespan reflects the dual role of the SDH complex: if SDH function is decreased, not only is the mitochondrial electron transport function damaged, but succinate-to-fumarate conversion in the TCA cycle is also impaired. Finally, Arg244His mutant worms display a rewired metabolism—an aberrant glycolysis reminiscent of the Warburg effect—which could explain the developmental differences between Arg244His animals and *sdhb-1* null mutants [159].

Lastly, *sdha-1* is the third member of the SDH complex that has also been characterized in worms. *sdha-1* loss-of-function mutant animals are viable but show slower movement and a reduced oxygen consumption rate and contain smaller and less networked mitochondria. Their development is slower from larval stages L2 to L3. Males cannot copulate due to an anal depressor muscle defect [162]. Goncalves et al. discovered metabolic changes in the absence of SDHA-1 function: the expression of phosphoenolpyruvate carboxykinase (PEPCK; *pck-1* and *pck-2,* parts of the gluconeogenesis pathway) is increased. In contrast, *sdha-1* overexpression has the opposite effect, suggesting that mitochondrial function and levels of anabolic processes are inversely correlated [162]. These the above observations illustrate multiple examples of metabolic adaptation—a hallmark displayed by tumors—in nematodes carrying different SDH deficiencies [163,164]. Altogether, *C. elegans* can reveal developmental abnormalities, metabolic changes, and oxidative effects caused by SDHx mutations.

### 4.3. Mutations Affecting CoQ Synthesis

It has been said that life depends on the stepwise transfer of electrons downhill through energy gradients. CoQ (Coenzyme Q/ubiquinone/ubiquinol) acts as transmitter of the electrons transferred from complexes I and II to complex III. The multistep synthesis of CoQ9 in *C. elegans* requires the activity of *coq-1*, *coq-2*, *coq-3*, *coq-8*, and *clk-1* (also called *coq-7*) genes. Knockout mutations in *coq-1*, *coq-2*, *coq-3*, and *coq-8* result in animals that can only survive for one generation on normal *E. coli* bacteria (*E. coli* strain OP50 produces CoQ8) but whose progeny become fertile [165,166,167,168]. Survival of the first homozygous generation is due to maternally deposited gene products and CoQ in the egg [169]. In contrast, *clk-1* (stands for clock) mutants can reproduce indefinitely on normal *E. coli* strain OP50 but cannot grow on bacteria that do not produce CoQ8 [170]. *clk-1* mutants accumulate biosynthetic intermediate demethylubiquinone-9 (DMQ9), which partially compensates for the absence of CoQ9 and allows these mutants to grow as long as dietary CoQ8 is available [170,171]. *clk-1* is actually one of the very first identified longevity genes (reviewed by Wang et al. [42]).

The two most studied mutations of *clk-1* are the *qm30* null allele and the *e2519* missense allele. All *clk-1* phenotypes are present in both mutants but are weaker in *e2519* mutants [172]: *clk-1* mutants show lifespan extension, reduced fecundity, decreased pharynx pumping, defecation, and locomotion, alongside slow embryonic and postembryonic development [172,173]. All *clk-1* mutant phenotypes (lifespan, behavioral phenotypes, and altered expression of mitochondrial quality control genes) were rescued by pharmacologically restoring CoQ biosynthesis, indicating that *clk-1* mutant phenotypes are entirely due to defects in CoQ biosynthesis [174]. *coq-3*(*ok506*) mutants have a shorter lifespan and retain fertility, but only a handful of the second-generation mutants develop to adulthood [168,175].

### 4.4. Mutations of Complex III Subunits and Cytochrome C

The *C. elegans* genome possess eight complex III subunit homologs: [131] among the three well characterized subunits, two are encoded in the nucleus (*isp-1*, the homolog of the mammalian Rieske iron sulfur protein and *cyc-1/*cytochrome C1), and one is encoded in the mitochondrial genome *ctb-1/*(cytochrome b). *isp-1*(*qm150*) mutants show “slow phenotypes”: they lay reduced numbers of eggs; have slower embryonic and postembryonic development and lower fecundity; and, importantly, live longer than wild-type animals [176]. Jafari et al. identified intragenic suppressors (such as A149T/V and A151T) in the highly conserved six-amino-acid tether region (DQRALA) of ISP-1 [177]. Suppressor mutants restored *isp-1*(*qm150*) phenotypes, including all “slow phenotypes” and lifespan extension. Furthermore, mutations analogous to Rieske protein Rip1 in budding yeast show similar effects, proving the conservation of this structure–function relationship across highly divergent species. *isp-1*(qm150) carries a proline-to-serine amino acid change at position 225, which lies in the head unit of ISP. Prolines are important structurally, as they make the peptide backbone rigid [178]. The ISP head region is a key element in ISP function. Based on multiple studies, the tether region seems to be a flexible element responsible for positioning the head region of ISP between three main locations: either to the electron donor or to the acceptor site or into an intermediate position [50,179]. In conclusion, mutations in the tether region are capable of compensating for the steric alteration of the ISP head caused by *isp-1*(*qm150*). Regarding lifespan phenotypes, similar to *isp-1*(*qm150*) mutation, RNAi against another complex III subunit *cyc-1* also extends lifespan [180]. This lifespan extension is suppressed by *icl-1*(*ok531*) due to the inactivation of the glyoxylate pathway [181]. It has been suggested that defective ISP function is compensated for by the expression of SOD3 (superoxide dismutase) [176] and ICL-1, the key enzyme of the glyoxylate shunt [182]. As expected, *isp-1*(*qm150*) mutants display a defective complex III function. The respiratory complexes can form higher-order assemblies called supercomplexes or respirasomes, consisting of monomeric CI and dimeric CIII and monomeric CIV [183]; it also was shown that complex I activity is not only impaired but that the I:III:IV supercomplex is also disrupted in these animals. It has been suggested that the assembly of supercomplexes may increase the efficiency of the electron transport chain, reducing the rate of ROS production [184]. Together, these data lead to the conclusion that ISP-1 may act as a stabilizer of the supercomplex through either protein–protein interactions or protein-conformational linkage [176].

Consistent with these data, *RNAi* specific for cytochrome C1 homolog *cyc-1* also extended lifespan [180], which was suppressed in the *icl-1*(*ok531*) mutant background, as a consequence of inactivation of the glyoxylate pathway [181].

Interestingly, a mutant allele of cytochrome b homolog *ctb-1*(*qm189*) suppresses the slow phenotypes mentioned above but not the elevated lifespan of *isp-1*(*qm150*) [176]. Feng et al. concluded that *ctb-1*(*qm189*) mutation has a double effect: on one hand, it causes loss of function in complex III, and on the other hand, possess gain-of-function in stabilizing the I:III:IV supercomplex, thereby compensating for the effects of *isp-1*(*qm150*).

CyC (cytochrome C) in *C. elegans* is encoded by two genes: *cyc-2.1.* and *cyc-2.2.* To date, these genes have not been deeply investigated. However, *C. elegans* CyC possesses similar properties as its mammalian homolog [185]. The germline serves as key tissue in lifespan regulation. *cyc-2.1* knockdown by RNAi in the germline extends lifespan by activating the intestinal mitochondrial unfolded protein response (UPRmt), AMPK (AMP-activated kinase), and mitochondrial fission [186].

### 4.5. Mutations of Complex IV and V Subunits

**Complex IV** (cytochrome C oxidase or COX) is regulated by signals of the intramitochondrial ATP/ADP ratio [187]. In electron transfer, complex IV involves two hemes (cytochrome a and cytochrome a3) and two copper centers (CuA and CuB). RNAi against COX-IV, COX-VB/*cco-*1, and COX-VA subunits decreases fecundity and slows development [188,189], while COX-VIIC (*RNAi*) causes arrest at the second larval stage [189]; in all cases, extended lifespan is observed [180,189].

Finally, **complex V** of the MRC is the ATP synthase, which is not directly involved in electron transport, unlike the other complexes. In *C. elegans*, the homologs of human ATP5B and ATP5O genes are integral to the known roles of mitochondrial dysfunction and aging. The *C. elegans* homolog of ATP5B, known as *atp-2*, is critical for the proper function of ATP synthase’s F1 subunit. *atp-2* (*ua2*) loss-of-function mutants have been demonstrated to result in reduced ATP synthesis, leading to decreased mitochondrial function and altered metabolic states that can accelerate aging or increase stress resistance, arrest at the third larval stage, and decreased pharynx pumping [131,135,190]. Similarly, the ATP5O homolog, *oscp-1*, plays a vital role in maintaining the stability and efficiency of ATP synthase. Studies have reported that disruptions in *oscp-1* (also known as *atp-3*) can lead to compromised energy production and are associated with phenotypes indicative of mitochondrial pathology. leading to effects such as reduced brood size, developmental delays, and increased sensitivity to mitochondrial toxins. Studies of *C. elegans* have also demonstrated that alterations in the homologous gene can disrupt the normal function and assembly of mitochondrial ATP synthase, reflecting phenotypic changes such as reduced fecundity, slower growth rates, and increased lifespan [180,191]. Retardation in gonadal development in response to *atp-3* silencing is the consequence of decreased ATP production, and it is known that germline development is very sensitive to available energy sources [192]. Xu et al. silenced *Y82E9BR.3*, the *C. elegans* ORF corresponding to human ATP synthase C subunit, which resulted in slowed development and sterility [193]. The *C. elegans* genome encodes two inhibitor proteins of the F1Fo-ATPase, MAI-1 and MAI-2. MAI-2 is localized to mitochondria: worms lacking MAI-2 function have an enhanced mitochondrial membrane potential and decreased physiological germ cell apoptosis, suggesting that MAI-2 might play a role in apoptosis by regulating mitochondrial membrane potential [194].

These studies highlight the immense importance of ATP synthase components in energy metabolism and stress response within *C. elegans*, demonstrating that such effects are not only observed in higher mammalians but genetic model systems like *C. elegans* that also exhibit a similar phenotype, thereby making this model a valuable tool for understanding the genetic and biochemical bases of mitochondrial pathologies.

## 5. *Caenorhabditis elegans* Serves as Test Bed for Drugs Acting on ETC Components

*C. elegans* can serve as a model system to test drugs acting on ETC components. For example, drug candidates inhibiting OXPHOS have emerged as promising targets of cancer therapeutics that have been tested on nematodes, focusing on lifespan effects. Antidiabetic agent metformin slows cancer progression both in vitro and in vivo by inhibiting mitochondrial complex I and increases ROS production with subsequent activation of HIF-1α [195]. Metformin extended lifespan in *C. elegans* [196]. Phenformin, another biguanide complex I inhibitor that was withdrawn from human use because of lactic acidosis in patients, showed promising results in combination with the chemotherapeutic agent gemcitabine in the treatment of high-OXPHOS pancreatic ductal adenocarcinoma models [197]. We note that phenformin also increased the lifespan of worms [198] and that Arctigenin (in Phase I trial), a lignan from *Arctium lappa.*, has been shown to extend lifespan, improve survival under oxidative stress, and decrease endogenous ROS levels in *C. elegans* [199]. Furthermore, some novel drugs targeting OXPHOS, such as GBS-01/Arctigenin, are in clinical trials [200].

Antibiotics have also been suggested in cancer therapy; for example, doxycycline was shown to act as a selective inhibitor of cancer stem cells [201,202]. Doxycycline also extends lifespan in *C. elegans* [203], reduces respiration, and activates mitochondrial unfolded protein response [204].

Aspirin’s well-known ability to reduce cancer risk [205] is understood to reduce oxidative stress and was reported to extend the lifespan of worms through a metabolic increase mediated by germline signaling [206].

Phenolic compounds synthesized by plants to increase their survival in response to environmental stresses are known to have antioxidative and anti-inflammatory effects. Resveratrol is a well-known example, which is present in grape, peanut, and berry fruits and associated with anti-obesity, cardioprotective, neuroprotective, antitumor, and antidiabetic properties [207]. Free-radical scavenging and antioxidative activities are well-known properties of resveratrol [208]. It is thought that many dietary compounds, including flavonols, function as antioxidants because they or their metabolites can alkylate Keap1. Keap1 alkylation leads to the activation of Nrf2 and its downstream target antioxidant enzymes [209]. The beneficial effects of resveratrol are also linked to mitochondrial biogenesis, and it has been suggested that resveratrol directly binds to complexes I [210], III [211], and V [212,213,214] and alters their activities (reviewed in [215]).

The antioxidant and ROS scavenging activities of resveratrol were tested by using free radical-producing chemicals juglone and paraquat as stressors in *C. elegans*. Worms treated with resveratrol under stressful conditions showed an expansion of lifespan [216]. The antioxidant curcumin (plant origin, widely used in Asia) has been used as a chemopreventive substance in cases of colorectal cancer [217]. Curcumin exerts its chemopreventive effect by inducing apoptosis of tumor cells through mitochondrially dependent pathways, such as loss of mitochondrial membrane potential, release of cytochrome C, changes in electron transport [218], activation of caspase pathways, and ROS production [219]. In addition, curcumin inhibits multiple cancer pathways [219]. Supplementation with curcumin caused an increased antioxidant capacity in worms and extended their lifespan [220]. Similarly, treatment with its derivative, curcumin–acetylsalicylate also resulted in lifespan extension [221].

## 6. Discussion

It has been suggested that ancient worms buried in the ocean floor, where sulfur–iron pyrites were abundant, may have sparked biodiversity [222]. Therefore, it may not be a coincidence that, to this day, FeS clusters are not only permissive for electron transport across species but also that the majority of human genes encoding ETC components have orthologs in *C. elegans* [131]. The worm provides an important test for human diseases of ETC function because ETC complex mutations affect all stages of *C. elegans* development, manifesting either developmental larval arrest, sterility, or reductions in lifespan. Table 1 summarizes human vs. nematode mutations in homologous genes encoding ETC complex subunits and their phenotypes. When comparing the type of mutant alleles in homologous genes, relatively few attempts have hitherto been made to reproduce the clinically relevant mutations in the nematode. Nonetheless, in three studies, mutant worms have recapitulated important aspects of the corresponding human disease. For example, worms carrying A352, T434M, and A443F substitutions in NDUFV1 homolog NUO-1 protein showed hallmarks of complex I dysfunction such as lactic acidosis and decreased NADH-dependent mitochondrial respiration. Furthermore, clinically relevant substitutions P211 and R244H in SDHB homolog SDHB-1 protein resulted in hypersensitivity to oxidative stress and aberrant, Warburg-like glycolytic activity, as also observed in SDHB mutant PHEO cell lines. In the future, genome editing techniques will allow for the generation of more mutants carrying clinically relevant mutations. Thus, worms can provide mechanistic insight.

Regarding similarities in the presentation of different ETC complex mutations in man and worm, we can see that both show measurable metabolic effects—notably, lactic acidosis. Human ACAD9 and COXVIA1 mutations manifest with muscle weakness; this phenotype has also been observed in several worm ETC mutants, such as *nuo-1*, *nuo-6*, *clk-1*, and *atp-2* mutants, which show slow locomotion, defecation defects, and decreased pharynx pumping due to neuromuscular impairment (see details in Table 1).

A pleiotropic phenotype, which can be the consequence of many developmental and signaling defects (therefore, hard to translate to human health), is arrest at the L3 larval stage that can be observed in several ETC mutants. L3-to-L4 transition has a high energy demand, accompanied by an increase in oxygen consumption [135]. Transition to the L4 stage is not possible in *nuo-1* and *atp-2* mutants because of their decreased energy production.

Aging phenotypes can help our understanding of age-related diseases, as *C. elegans* has been extensively used as an aging model [223]. Indeed, many signaling pathways that regulate aging were first described in the worm, for example, insulin signaling or the mTOR pathway. Together, numerous genetic and environmental conditions are known to regulate aging, as reviewed elsewhere [224]. Loss of mitochondrial function is a hallmark of aging, as the ETC is impaired in old age [225]. The detrimental effects of different ETC mutations on lifespan have been widely studied in worms. Complex II mutations shorten the lifespan of worms, which reflects the dual role of the SDH complex: the improper function of the SDH complex results in a hindered succinate-to-fumarate conversion in the TCA-cycle, as well as a damaged mitochondrial electron transport function. Mutations in other complex components affect lifespan differently: when mutated or impaired, some complex I, III, and IV defects shorten the lifespan (for example, *gas-1*(*fc21*)); others, in contrast, extend it (*nuo- 1*(*ua1*), *nuo-6*(*qm200*), *clk-1*(*e2519*), *isp-1*(*qm150*), and *atp-2*(*ua2*)). Actually, the majority of mutations in genes encoding complex I, III, and V subunits, as well as RNAi specific for complex IV subunits, result in lifespan extension. Extension of lifespan was also observed in *Drosophila* when five genes encoding components of respiratory complexes I, III, IV, and V were treated by RNAi [226]. Interestingly, in mice, mutation of complex IV assembly factor *Surf1* and heterozygous mutations in *MCLK1*, a factor involved in the synthesis of ubiquinone, increase lifespan [227,228].

One model to explain the lifespan-extending effect of ETC mutations in several genetic models is the “rate of living” hypothesis, originally proposed by Max Rubner in 1908, which argues that a lower basal metabolic rate caused by decreased ETC function increases life expectancy [229].

However, increased lifespan does not automatically mean a longer healthy life [230]. Analysis of healthspan is also possible in *C. elegans* by using different parameters in aging worms. For example, a study by Bansal et al. [231] examined four parameters, namely resistance to heat and oxidative stress and movement capacity in liquid and solid media in long-lived mutants, including *clk-1*(*qm30*)*. clk-1* mutants animals showed extended lifespan but not healthspan, as they exhibited an increased ratio of time spent in a frail state.

It is intriguing that mutations in genes encoding subunits of complex I and complex III have been reported to increase lifespan in worms in a ROS-dependent manner [141]. Increased longevity observed in *nuo-6* and *isp-1* partial loss-of-function mutants is due to elevated superoxide levels [232]. It was also shown that elevated ROS levels in *isp-1* mutants cause the activation of stress response pathways such as the mitochondrial unfolded protein response, the SKN-1-mediated stress response, and the hypoxia response [233].

This idea is consistent with both hormesis and eustress, where a transient increase in ROS levels activates a stress response pathway. Complex I mutations can also extend the fly lifespan through a similar mechanism [234]. These discoveries underpin the concept of mitohormesis, which proposes that boosting ROS levels activates a stress response to compensate for initial damage (reviewed in [83]). Metformin, a non-specific inhibitor of complex I also extended lifespan of worms, also through a mitohormetic response: the metformin–ROS signal activated the PRDX-2 periredoxin pathway, which led to lifespan extension [235].

Although in vitro measurements have identified eleven ROS-generating sites on isolated mitochondria (associated with substrate catabolism and the ETC [236]), the main mtROS generators are complex I and complex III of the ETC. To better understand the role of complex I- and complex III-derived ROS in different physiological and pathological processes, recent research has focused on specific sites, where electrons leak, for example, the flavin site (I_F_) and quinone binding site (I_Q_) of complex I (reviewed in [237]). Certain conditions induce the production of ROS at the I_Q_ site by reverse electron transport (RET). Better understanding of the regulation of RET-ROS is crucial, as it not only contributes to different physiological processes but is also linked to stroke and age-related diseases.

In *C. elegans*, RET was induced by inhibiting complex V using TCA-cycle metabolite alpha-ketoglutarate. Interestingly, alpha-ketoglutarate administration in worms resulted in lifespan extension, which is dependent on mTOR, although the exact mechanism has not been described [238]. ROS produced by RET has also been shown to extend lifespan in *Drosophila* [118].

To comprehend the role of mtROS in health and disease and potentially use it as a therapeutic tool in the future, we need to understand where (in which site) and when ROS are generated and how the intensity of the ROS signal influences the outcome. A recent study in *C. elegans* showed that elevating ROS levels experimentally during the L2 larval stage of development results in lifespan extension, meaning that exposure to oxidants during early development influences the duration of the adult lifespan [239]. These data also show that *C. elegans*, among other genetic models, continues to help our understanding of spatio-temporal contexts of ROS signaling.

Together, the above data demonstrate that *C. elegans* will continue to serve as a tractable genetic model in further understanding of signaling pathways both up- and downstream of ROS. In addition, this tractable nematode has emerged as an alternative platform in drug testing; thus. compounds acting on the ETC and/or influencing mtROS levels can also be examined using worms.

## 7. Conclusions

Mutations in highly conserved genes encoding components of the electron transport chain (ETC) provide valuable insights into the mechanisms of oxidative stress and mitochondrial ROS (mtROS) in a wide range of diseases, including cancer, neurodegenerative disorders, and aging. *C. elegans* provides an important test for human diseases of ETC function because ETC complex mutations affect all stages of worm development, manifesting either developmental larval arrest, sterility, or reductions/extensions in lifespan. Some studies have reproduced clinically relevant mutations in homologous complex I or complex II ETC subunits in *C. elegans*, and the mutant worms recapitulated human metabolic phenotypes such as lactic acidosis or aberrant, Warburg-like glycolytic activity. In the future, genome editing techniques will allow for the generation of more mutants carrying clinically relevant mutations; thus, worms can provide mechanistic insights.

Aging phenotypes can help our understanding of age-related diseases, as *C. elegans* has been extensively used as an aging model; however, increased lifespan does not automatically mean a longer healthy life, which is a challenging task to address. Analysis of healthspan is also possible in *C. elegans* by using different parameters in aging worms.

Although in vitro measurements have identified eleven ROS-generating sites on isolated mitochondria, the main mtROS generators are complex I and complex III of the ETC. It is intriguing that mutations in genes encoding subunits of complex I and complex III have been reported to increase lifespan in worms in an ROS-dependent manner. This idea is consistent with both hormesis and eustress, where a transient increase in ROS levels activates a stress response pathway. These discoveries underpin the concept of mitohormesis, which proposes that boosting ROS levels activates a stress response to compensate for initial damage.

*C. elegans* has great potential as a platform for testing ETC-targeting drug candidates, including OXPHOS inhibitors, which represent promising avenues in cancer therapeutics. Together, these data demonstrate that *C. elegans* will continue to serve as a tractable genetic model in further understanding of signaling pathways both up- and downstream of ROS.

## Figures and Tables

**Table 1:** Phenotypic outcomes of ETC dysfunction-related loss-of-function mutations in *C. elegans,* focusing on lifespan alterations. Regarding the human clinically relevant mutations, in this table, we presented some representative mutations.

						Mutant Phenotypes				
Complex	Human Subunit	Human Clinical Mutations *	Human Phenotypes	*C. elegans* Homolog	Allele/Type of Mutation	Larval Arrest	Germline Defect	Lifespan	ROS Level	Other
Complex I	NDUFV1	More than 40 substitutions have been identified	Leigh syndrome, Leigh-like syndrome, hypotonia, lethargy, myopathy, and fatigue	*nuo-1*	ua1/deletion	L3	Arrest of gonad development at L2	Increased		Impaired mobility, pharyngeal pumping, and defecation
		A341V, T423M, and A432F	Leukodystrophy and myoclonic epilepsy	*nuo-1*	A352V, T434M, and A443F			Reduced		They all display increased lactate and lactate:pyruvate ratios, low brood size, and hypersensitivity to hyperoxia and paraquat, and the A352V mutant animals develop slower
				*nuo-1*	*RNAi*					Acidosis
	NDUFS2	E104A, F84L, M292T, R118Q, M443K, E148K, R138Q, R333Q, R228Q, P229Q, and S413P	Leigh syndrome, Leigh-like syndrome, cardiomyopathy, and encephalomyopathy	*gas-1*	fc21/deletion	-		Reduced		Slowed development and hypersensitivity to volatile anesthetics
	NDUFB4	N24A and R30A	Disruption of supercomplex assembly	*nuo-6*	*qm200/* substitution	-		Increased	Slightly decreased global ROS generation but significantly increased superoxide generation	Decreased respiration and electron transport rates and slower embryonic and postembryonic development
	NDUFA6	Arg64Pro, Glu89∗, Glu111Serfs∗35, c.3G > A, Met104Cysfs∗35, and Leu119Tyrfs∗20	Neuroradiological findings and/or elevated lactate levels	*nuo-3*	*G60D*	-				Restores complex I activity of *nduf-7*(*et19*) and *gas-1*(*fc21*) mutants
	ACAD9	Deletions, Val546Leu, Ala170Val, His563Asp, Arg414Ser, and Leu558Profs*45	Heart, muscle, liver, and nervous system disorders	*acdh-12*	*RNAi*			Reduced		Decreases fecundity
Complex II	SDHA	Arg31*, L511P, G233V, Arg512*, S445L, and UTRdel	PHEO/PGL and GIST	*sdha-1*	*rg550/* substitution					Slower movement, development from L2 to L3, egg retention, and partial resistance to ROS-producing poison paraquat
	SDHB	Arg27*, Arg46Gly, Arg90*, and 311delAinsGG	PHEO/PGL, GIST, renal cell carcinoma, multiple hamartomas, and T-cell acute leukemia	*sdhb-1*	*gk165/*deletion	L2/L3	Arrest of gonad development at late L1	Reduced		
		Arg230His	PHEO/PGL	*sdhb-1*	Arg244His	-	Sterile, incompletely developed gonad arms	Reduced		Sterile, aberrant glycolysis
		Pro197	PHEO/PGL	*sdhb-1*	Pro211 mutants		-	Reduced	Increased	Premature aging, embryonic lethality, and hypersensitivity to oxidative stress
	SDHC	Deletion and many substitutions, the most common of which is Arg133 affection	*PGL*	*mev-1*	*kn1* substitution	-	-	Reduced	Increased	Paraquat sensitivity and reduced brood size
				*mev-1*	*tm1081/*deletion					Lethal/sterile
Coenzyme Q	COQ3			*coq* *-3*	*qm188/*deletion	L1 (homozygous mother)	Sterile (heterozygous mother)	Reduced	-	
				*coq-3*	*ok506/*deletion		Gonads appear abnormal	Reduced		
	COQ7	R54Q, 1Met?, Ala205HisfsTer48, Met135Val, and Pro108Thr	Decrease in coenzyme Q10 production, hereditary motor neuropathy, cardiomyopathy, gastrointestinal obstruction, and hereditary spastic paraplegia	*clk-1*	*qm30/*deletion	-		Increased	-	Slow embryonic and postembryonic development; reduced fecundity, defecation, and locomotion; decreased pharynx pumping; and altered expression of mitochondrial quality control genes
				*clk-1*	*qm51/*aberrant intron 2 splicing and early stop codon			Increased	-	Slow embryonic and postembryonic development; reduced fecundity, defecation, and locomotion; decreased pharynx pumping; and altered expression of mitochondrial quality control genes
				*clk-1*	*e2519/*missense	-		Increased	-	Slow embryonic and postembryonic development; reduced fecundity, defecation, and locomotion; decreased pharynx pumping; and altered expression of mitochondrial quality control genes
Complex III	UQCRFS1	Val72_Thr81del10 and combination of Val14Asp and Arg204 *	Decreased complex III activity, cardiomyopathy, and alopecia totalis	*isp-1*	*qm150/*substitution	-		Increased	Decreased	Slow phenotypes
	CYC1	Trp96Cys and Leu215Phe	Insulin-responsive hyperglycemia	*cyc-1*	*RNAi*			Increased		
Cytochrome C	CYCS	Lys101del, His27Tyr, G41S, Y48H, and Tyr98His	Thrombocytopenia, non-syndromic thrombocytopenia, and non-syndromic thrombocytopenia	*cyc-2.1*	*RNAi*			Increased	-	Activates AMPK, mitochondrial fission, and UPR^mt^
Complex IV	COXIV	K101N, P152T, and E138K	Resembles Fanconi anemia and Leigh syndrome; causes calvarial hyperostosis and dyserythropoietic anemia	*cox-4*	*RNAi*	-		Increased		Decreased fecundity and slowed development
	COXVA	R107C	Lactic acidemia and pulmonary arterial hypertension	*cox-5A*	*RNAi*	-		Increased		Decreased fecundity and slowed development
	COXVB			*cox-5B/cco-1*	*RNAi*	-		Increased		Decreased fecundity, slowed development, and induction of UPR^mt^
	COXVIIC			*cox-7C*	*RNAi*	L2		Increased		
Complex V	ATP5B	Thr334Pro and Val482Ala	Dominantly inherited dystonia	*atp-2*	*ua2/*deletion	L3	Arrest of gonad development at L2	Increased	-	
	ATP5O			*atp-3*	*RNAi*		Retardation in gonad maturation	Increased	-	

UPR^mt^: mitochondrial unfolded protein response. * Due to space limitations, only a few examples are presented in the table. For detailed descriptions, please see the text.

## Data Availability

Not applicable.
